# Patient-adapted organ absorbed dose and effective dose estimates in pediatric 18F-FDG positron emission tomography/computed tomography studies

**DOI:** 10.1186/s12880-020-0415-4

**Published:** 2020-01-29

**Authors:** Brian M. Quinn, Yiming Gao, Usman Mahmood, Neeta Pandit-Taskar, Gerald Behr, Pat Zanzonico, Lawrence T. Dauer

**Affiliations:** 1 Department of Medical Physics, Memorial Sloan Kettering Cancer Center, 1275 York Avenue, New York, NY 10065 USA; 2 Department of Radiology, Memorial Sloan Kettering Cancer Center, 1275 York Avenue, New York, NY 10065 USA

**Keywords:** PET/CT dosimetry, CT dosimetry, PET dosimetry, Effective dose, Pediatric

## Abstract

**Background:**

Organ absorbed doses and effective doses can be used to compare radiation exposure among medical imaging procedures, compare alternative imaging options, and guide dose optimization efforts. Individual dose estimates are important for relatively radiosensitive patient populations such as children and for radiosensitive organs such as the eye lens. Software-based dose calculation methods conveniently calculate organ dose using patient-adjusted and examination-specific inputs.

**Methods:**

Organ absorbed doses and effective doses were calculated for 429 pediatric 18F-FDG PET-CT patients. Patient-adjusted and scan-specific information was extracted from the electronic medical record and scanner dose-monitoring software. The VirtualDose and OLINDA/EXM (version 2.0) programs, respectively, were used to calculate the CT and the radiopharmaceutical organ absorbed doses and effective doses. Patients were grouped according to age at the time of the scan as follows: less than 1 year old, 1 to 5 years old, 6 to 10 years old, 11 to 15 years old, and 16 to 17 years old.

**Results:**

The mean (+/− standard deviation, range) total PET plus CT effective dose was 14.5 (1.9, 11.2–22.3) mSv. The mean (+/− standard deviation, range) PET effective dose was 8.1 (1.2, 5.7–16.5) mSv. The mean (+/− standard deviation, range) CT effective dose was 6.4 (1.8, 2.9–14.7) mSv. The five organs with highest PET dose were: Urinary bladder, heart, liver, lungs, and brain. The five organs with highest CT dose were: Thymus, thyroid, kidneys, eye lens, and gonads.

**Conclusions:**

Organ and effective dose for both the CT and PET components can be estimated with actual patient and scan data using commercial software. Doses calculated using software generally agree with those calculated using dose conversion factors, although some organ doses were found to be appreciably different. Software-based dose calculation methods allow patient-adjusted dose factors. The effort to gather the needed patient data is justified by the resulting value of the characterization of patient-adjusted dosimetry.

## Background

About half of the ionizing radiation exposure to the United States population is estimated to be from medical imaging procedures, including radiopharmaceutical imaging and computed tomography [1]. Through the combination of physiological information from positron emission tomography (PET) and anatomical information from computed tomography (CT), PET-CT has become established prominently in the diagnosis and treatment monitoring of many types of cancer. The sequential acquisition of PET and CT images in 2–18-Fluoro-2-deoxy-D-glucose (18F-FDG) PET-CT results in patient radiation dose from both imaging modalities but the risk incurred from this radiation dose is generally thought to be justified by the benefit of the diagnostic information obtained from the scan. Previous studies of 18F-FDG PET-CT dosimetry have reported adult effective dose (E) of 13 to 32 mSv and pediatric (E) of 7 to 29 mSv [2–6]. The wide range of reported PET-CT effective doses reflects varying conventions and technical parameters of use of CT in PET-CT examination, variations in injected 18F-FDG activity, range of patient age and body sizes as well as varying dosimetry methodologies. With radiation dose from each scan and multiple scans over the course of disease management, optimization of radiation dose in 18F-FDG PET-CT is especially important for children, who have longer life expectancy over which to undergo multiple scans and are generally thought to be more radiosensitive than adults [7]. While E is useful for comparison of ionizing radiation procedures, it should not be used to assess individual detriment and is used most appropriately in radiation protection for setting secondary limits for intakes of radionuclides and for ensuring that exposure limits for radiation workers are not exceeded [8, 9]. Tissue weighting factors, based on population-averaged values, as used in the calculation of E, make E no more a reliable indicator of individual detriment than population-based organ-specific factors [10]. In the current paradigm of radiation protection, the known relationship between dose and risk at higher radiation dose is assumed to extrapolate linearly to that at lower dose, and children are considered to be at greater risk of developing radiation-induced tumors due to their life expectancy and higher radiosensitivity of select tissues [7, 10–12]. The basis for the belief of relatively higher risk for children demonstrated in a report by the National Research Council is challenged by some in light of their view that the risks at low radiation doses such as those incurred during medical imaging procedures are not unequivocally supported by current epidemiological data [13, 14]. The limitations of popular approaches to risk quantification are widely recognized. In their overview of the debate surrounding the use of the linear no-threshold dose-response model, Zanzonico and Weber acknowledge that the uncertainty in correlation between diagnostic radiation dose and detriment propagates to the process of making clinical decisions for individual patients [15].

Despite debated cogency of linear extrapolation of risk from known, higher doses to that at diagnostic imaging levels, and despite critical acceptance of the relative radiosensitivity of the pediatric population, various ongoing efforts attempt to optimize and limit pediatric medical imaging radiation dose. The Image Gently Alliance advocates for safe and effective imaging care of children and raising awareness in the imaging community of the need to adjust radiation dose when imaging children [16]. The Image Gently campaign addressed radiation dose from both CT and PET scans through guidelines brought forth by founding and alliance organizations. Image Gently maintains published suggestions for either developing CT protocols for children or verifying that current pediatric protocols are appropriate, and the Alliance for Quality Computed Tomography of the American Association of Physicists in Medicine (AAPM) has developed reference pediatric CT protocols [17, 18]. The American College of Radiology (ACR) introduced the CT Dose Index Registry in 2011 to facilitate the collection and comparison of CT dose indices, although pediatric data are not currently included [19]. In 2008 the European Association of Nuclear Medicine (EANM) first published suggested pediatric nuclear medicine injected activities, and in 2011 the North American consensus guidelines recommended a similar set of administered activities for pediatric nuclear medicine. The pediatric radiopharmaceutical administered activity currently recommended by Image Gently is based on the 2016 update to the North American Consensus Guidelines and notes the EANM dosage card may also be used for some radiopharmaceuticals [20]. Such efforts to make available typical radiation doses and standardize some aspects of pediatric medical imaging provide a framework for optimization, with the intent that patient radiation dose is minimized while maintaining diagnostic utility of the resulting images. In previous studies of optimization of pediatric PET-CT, including non-18F-FDG PET-CT, other authors recognize the contribution of both modalities to total patient radiation dose and the authors reveal opportunities to optimize aspects of both [21, 22]. For example, patient preparation, immobilization, use of recommended administered activities, and careful selection of CT protocol all ensure image quality while optimizing patient radiation [23]. In an exploration of operational and dosimetric aspects of pediatric PET-CT, the challenges of imaging children are recognized along with optimization opportunities, with an emphasis on the importance of understanding the role of CT in this examination [24]. CT technique is chosen based on the objective of the examination, which may require high-resolution delineation of organs, bones, soft tissue or blood vessels. In the case of PET-CT, x-rays from CT are used to construct an attenuation map of density differences throughout the body that can then be used to correct for the absorption of the photons emitted from 18F decay. This process of so-called CT attenuation correction (CTAC) is indirectly related to image formation and delivers less radiation dose than a CT technique intended to primarily provide images with useful diagnostic information. Previous studies have reported adult CT E from CTAC-only as 1.3 to 4.5 mSv, and one estimate of pediatric diagnostic whole-body CT E as high as 28 mSv [21, 25, 26].

Dose estimation methodology itself is integral to optimization and understanding the role of the many factors contributing to patient radiation dose in medical imaging [27]. A dosimetry method may consist of a dose coefficient applied to an examination-specific parameter such as injected activity or may employ computer simulation data based on a simplistic or anatomically realistic phantom. CT radiation dose, for example, may be estimated based on a singular CT dose metric or a computer simulation of the radiation from the specific CT model and using an anatomically realistic phantom. The latest versions of commercially available internal dose estimation software remain rooted in the methodology developed by the Medical Internal Radiation Dose (MIRD) Committee of Society of Nuclear Medicine and Molecular Imaging and offer a choice of many anatomically realistic phantoms with the latest tissue weighting factors, while reporting both organ and E for many radionuclides [28]. Likewise, modern CT dosimetry software is based on a comprehensive database of organ doses derived from Monte Carlo simulations involving a library of anatomically realistic phantoms [29]. A dosimetry method utilizing exam-specific information is more precise and therefore more valuable than one that does not. In this sense, the investigation of results utilizing the latest methodology is a pursuit of more valuable information.

The purpose of this study was to take advantage of the pediatric oncology patient population at our institution and available dosimetry software to evaluate a large pediatric patient cohort with patient-adjusted information. Patient-adjusted organ dosimetry of pediatric oncology patients undergoing 18F-FDG was performed utilizing patient-size parameters, individual injected activity and actual scan parameters. The PET portion was evaluated using OLINDA/EXM version 2.0 (OLINDA 2.0, Vanderbilt University), while the CT portion was evaluated using VirtualDose CT (Virtual Phantoms, Inc.). The results of this study are useful to evaluate the practicality of these methods and to characterize our patient population and reveal opportunities for optimization.

## Methods

Organ absorbed doses and E were calculated for 429 pediatric 18F-FDG PET-CT examinations performed over a 2-year period, comprised of 198 unique patients. A waiver of informed consent was obtained from the Institutional Review Board for retrospective review of patient data. GE DoseWatch software (GE Healthcare, Waukesha, WI) was used to identify pediatric PET-CT protocols in the desired examination date range. The following patient-adjusted and examination-specific information was recorded from the patient medical record and CT dose monitoring software: Age at scan time, gender, body mass, injected activity (MBq), x-ray tube voltage (kVp), x-ray tube current (mA), mAs/rotation, mAs-normalized weighted CT dose index (CTDIw/100 mAs), pitch, and volume CT dose index (CTDIvol). 18F-FDG activity to be administered (A_inj_) was determined for pediatric patients as the ratio of patient body surface area (BSA_ped_, m^2^) to that of adult body surface area, multiplied by the nominal adult injected activity of 12 mCi (444 MBq) [30].

$$ {\mathrm{A}}_{\mathrm{inj}}\ \left(\mathrm{MBq}\right)=\left({\mathrm{BSA}}_{\mathrm{ped}}/1.77\right)\ast 444 $$

Injected activity is summarized in Table 1.

**Table 1:** Injected Activity

	All ages	< 1 Y	1–5 Y	6–10 Y	11–15 Y	16–17 Y
N	429	3	60	118	167	81
Mean Injected 18F-FDG Activity (MBq)	300	96	178	282	389	427
Standard Deviation	104	8.2	35.5	61	50.6	28.6
Min	87.3	87	122	200	285	374
Max	477.3	104	237	477	477	477

<1Y = patients aged less than one year at scan time

1-5Y = patients aged one year to five years old at scan time

6-10Y = patients aged five years to ten years old at scan time

11-15Y = patients aged eleven to fifteen years old at scan time

16-17Y = patients aged sixteen to seventeen years old at scan time

Patients were divided into five groups according to age at the time of the examination: less than 1 year old (< 1), one to 5 years old (1–5), six to 10 years old (6–10), 11 to 15 years old (11–15), and 16 to 17 years old (16, 17).

A summary of patient body masses is presented in Table 2.

**Table 2:** Patient body mass

	All ages	< 1 Y	1–5 Y	6–10 Y	11–15 Y	16–17 Y
N	429	3	60	118	167	81
Median Body Mass, kg	32.3	7.5	15.7	27.4	48.3	62.3
Standard Deviation	19.2	0.2	4.5	11.9	12.1	11.6
Min	7.1	7.1	9.9	19.4	35	45.4
Max	87	7.5	27.2	68	77.6	87

<1Y = patients aged less than one year at scan time

1-5Y = patients aged one year to five years old at scan time

6-10Y = patients aged five years to ten years old at scan time

11-15Y = patients aged eleven to fifteen years old at scan time

16-17Y = patients aged sixteen to seventeen years old at scan time

All PET-CT examinations were performed with a GE Discovery 690 PET-CT, the CT portion comprised of a GE Lightspeed 16 CT unit.

The PET scan technique for all patients was a whole-body 3D PET protocol. The CT scan technique for all patients was an attenuation correction/localization (ACL) scan using a tube voltage and current selected based on patient body mass. Pitch factor was 0.98 or 1.38, rotation time 0.5 or 0.8 s, and tube potential 100 or 120 kVp. The tube current was specified according to body mass: less than 40 kg, 40 mA; 41–60 kg, 60 mA; 61–80 kg, 70 mA; 80–100 kg, 85 mA; and greater than 100 kg, 100 mA. A “scout” scan was performed at 10 mA prior to the ACL scan for gross anatomical visualization.

OLINDA/EXM Version 2.0 (Vanderbilt University) was used to calculate PET organ radiation absorbed doses and ED. The program requires specification of the radionuclide, organ residence times, and anatomic phantom. The program offers the choice of twenty-five human and ten animal (rodent) phantoms. The phantoms chosen for the current study include male or female newborn, 1-year-old, 5-year-old, 10-year-old, 15-year-old and adult. 18F-FDG residence times defined in ICRP 128 were used as input to the software [31]. Phantom was chosen by matching patient mass to the closest phantom mass, and phantom organ masses were scaled in the program by the ratio of the patient mass to the phantom mass. The program then produced dose factors for each organ, in terms of equivalent dose and E per unit injected activity (mSv/MBq). The dose factors were multiplied by the injected activity to obtain the total equivalent dose for each defined organ and the total E.

While the program produced factors of equivalent dose as mSv, due to the fact that 1 mSv is equal to 1 mGy for the radiations of concern, organ radiation absorbed dose is reported in Table 3 as mGy. Total colon dose was calculated by averaging the reported dose to the left colon, right colon and rectum.

**Table 3:** PET Organ Absorbed Dose (mGy)

	All ages	< 1 Y	1–5 Y	6–10 Y	11–15 Y	16–17 Y
Adrenals	7.1	8.1	7.7	7.4	6.6	6.2
Brain	11.2	6.8	8.0	10.6	14	15
Breast	4.2	N/A	N/A	N/A	4.0	4.3
Colon	7.4	8.0	7.8	7.7	6.9	6.4
Esophagus	7.9	9.4	8.8	8.3	7.1	6.6
Eyes	5.7	6.3	5.9	5.9	5.5	5.3
Gall Bladder	6.9	7.6	7.2	7.2	6.5	6.2
Gonads	6.9	7.6	7.6	7.1	6.5	6.0
Heart	42	38	38	48	42	35
Kidneys	6.3	7.4	6.9	6.5	5.6	5.3
Liver	12	12	12	13	12	11
Lungs	11	13	12	12	10	10
Pancreas	7.3	8.4	7.9	7.6	6.6	6.2
Prostate/Uterus	10	9.4	10	11	10	8.5
Red Marrow	4.8	7.1	4.7	4.8	4.6	4.7
Small Intestine	6.9	8.1	7.6	7.2	6.2	5.7
Spleen	6.1	7.2	6.8	6.3	5.5	5.1
Stomach	6.6	7.6	7.2	6.9	6.1	5.7
Thymus	7.5	8.4	7.9	7.7	7.0	6.6
Thyroid	6.2	7.4	6.9	6.5	5.5	5.0
Bladder	49	46	54	51	46	42

<1Y = patients aged less than one year at scan time

1-5Y = patients aged one year to five years old at scan time

6-10Y = patients aged five years to ten years old at scan time

11-15Y = patients aged eleven to fifteen years old at scan time

16-17Y = patients aged sixteen to seventeen years old at scan time

*N/A* Not applicable, PET dose not calculated for these phantoms

VirtualDose CT (Virtual Phantoms, Inc.) was used to calculate CT organ absorbed doses and E. VirtualDose CT offers 23 phantoms and the phantoms used in this study were male and female newborn, 1-year-old, 5-year-old, 10-year-old, 15-year-old and adult. The phantom was chosen by matching patient mass to the closest phantom mass. CT absorbed dose (mGy) was reported by VirtualDose CT for the organs and tissues in Table 4. Breast dose is only reported in phantom age 15-year-old and older, breast dose reported in the table is gender averaged. Total colon dose was calculated by averaging the reported dose to the colon and rectosigmoid colon. The software also reported total E utilizing tissue weighting factors in Report 103 of the International Commission of Radiological Protection. Eye lens dose was among those reported by this software. Scan range was selected within the software to indicate the head-to-toe scan range used for all pediatric PET-CT protocols.

**Table 4:** CT Organ Absorbed Dose (mGy)

	All ages	< 1 Y	1–5 Y	6–10 Y	11–15 Y	16–17 Y
Adrenals	5.9	4.5	5.6	5.8	6.0	7.0
Brain	6.1	4.3	5.3	5.9	6.6	7.8
Breast	5.4	4.0	5.4	5.5	5.3	5.9
Colon	6.7	4.8	6.4	6.8	6.8	7.3
Esophagus	6.4	5.0	6.2	6.3	6.2	7.4
Eyes	6.7	4.5	5.8	6.7	7.5	9.2
Eye Lens	7.0	4.1	5.9	7.0	8.0	9.9
Gall Bladder	6.0	4.6	5.9	6.2	5.9	5.9
Gonads	6.9	5.4	6.9	7.2	6.9	6.7
Heart	6.7	5.3	6.5	6.8	6.6	7.2
Kidneys	7.0	5.4	6.8	7.1	6.9	7.5
Liver	6.5	4.9	6.2	6.6	6.5	7.0
Lungs	6.6	5.3	6.4	6.7	6.6	7.2
Pancreas	6.0	4.8	6.0	6.2	5.9	6.3
Prostate/Uterus	6.1	4.6	6.1	6.2	5.9	6.4
Red Marrow	5.6	4.2	5.4	5.9	5.7	5.7
Small Intestine	6.3	4.7	6.1	6.4	6.3	6.9
Spleen	6.5	5.1	6.2	6.5	6.5	7.0
Stomach	6.3	4.8	6.1	6.5	6.6	6.4
Thymus	9.4	7.0	9.1	9.7	9.5	9.6
Thyroid	9.4	5.8	8.2	8.8	9.8	13.0
Bladder	6.6	5.0	6.6	6.8	6.5	6.9

<1Y = patients aged less than one year at scan time

1-5Y = patients aged one year to five years old at scan time

6-10Y = patients aged five years to ten years old at scan time

11-15Y = patients aged eleven to fifteen years old at scan time

16-17Y = patients aged sixteen to seventeen years old at scan time

The body masses of the phantoms used in VirtualDose CT and OLINDA are shown in Table 5.

**Table 5:** Phantom Masses, kg

	VirtualDose	OLINDA/ICRP 89^a^
Newborn	3.27	3.5
1 y/o	9.39	10
5 y/o	16.45	19
10 y/o	30.16	32
15 y/o M	53.13	56
15 y/o F	52.24	53
A M	73	73
A F	60.1	60

1 y/o = 1-year-old phantom

5 y/o = 5-year-old phantom

10 y/o = 10-year-old phantom

15 y/o M = 15-year-old phantom, male

15 y/o F = 15-year-old phantom, female

A M = Adult male phantom

A F = Adult female phantom

^a^OLINDA/EXM V2.0 uses ICRP 89 phantom masses

For both PET and CT dose, genitourinary organ dose is reported as prostate for male and uterus for female. Gonad dose is estimated as testes for male and ovaries for female, and the gender average gonad dose is reported in the tables. Total organ radiation absorbed dose to a given organ was calculated as the sum of the doses from PET and from CT for that organ, as shown in Table 6.

**Table 6:** PET + CT Total Organ Absorbed Dose (mGy)

	All ages	< 1 Y	1–5 Y	6–10 Y	11–15 Y	16–17 Y	
Adrenals	13	13	13	13	13	13	
Brain	17	11	13	17	21	23	
Breast	6.1	4.0	5.4	5.5	6.9	8.4	
Colon	14	13	14	14	13	13	
Esophagus	14	14	15	15	13	14	
Eyes	12	11	12	13	12	8.7	
Eye Lens	7.0	4.1	5.9	7.0	8.0	9.9	
Gall Bladder	13	12	13	13	12	12	
Gonads	14	13	14	14	13	13	
Heart	49	44	45	55	49	42	
Kidneys	13	13	14	14	13	13	
Liver	19	17	18	19	18	18	
Lungs	18	18	19	18	17	17	
Pancreas	13	13	14	14	13	12	
Prostate/Uterus	16	14	16	17	16	15	
Red Marrow	10	11	10	11	10	10	
Small Intestine	13	13	14	14	12	13	
Spleen	13	12	13	13	12	12	
Stomach	13	12	13	13	13	12	
Thymus	17	15	17	17	16	16	
Thyroid	16	13	15	15	15	18	
Bladder	56	51	60	57	52	49	

<1Y = patients aged less than one year at scan time

1-5Y = patients aged one year to five years old at scan time

6-10Y = patients aged five years to ten years old at scan time

11-15Y = patients aged eleven to fifteen years old at scan time

16-17Y = patients aged sixteen to seventeen years old at scan time

## Result

Table 6 presents the total (PET + CT) organ radiation absorbed dose for each age group, Table 3 presents PET organ radiation absorbed dose for each age group, and Table 4 presents CT organ radiation absorbed dose for each age group. A summary of calculated effective doses is presented in Table 7.

**Table 7:** Effective Dose (mSv)

	All ages	< 1 Y	1–5 Y	6–10 Y	11–15 Y	16–17 Y
PET						
mean	8.1	8.6	8.7	8.2	7.7	7.3
SD	1.2	0.8	1.5	1.1	0.5	0.9
Range	5.7–16.5	7.8–9.4	6.9–16.5	6.1–12.2	6.8–8.4	5.7–9.2
mean	6.4	4.8	6.2	6.5	6.4	6.9
CT						
SD	1.8	1.1	1.9	2.4	1.0	1.0
Range	2.9–14.7	4.1–6.1	2.9–14.7	3.6–14.2	4.2–7.8	5.2–9.1

SD = Standard Deviation

<1Y = patients aged less than one year at scan time

1-5Y = patients aged one year to five years old at scan time

6-10Y = patients aged five years to ten years old at scan time

11-15Y = patients aged eleven to fifteen years old at scan time

16-17Y = patients aged sixteen to seventeen years old at scan time

The five organs with highest total dose from PET and CT combined, as well as for PET alone were: Urinary bladder, heart, liver, lungs, brain.

The five organs with highest CT dose were: Thymus, thyroid, kidneys, eye lens, gonads (testes, male; ovaries, female).

For all patients, the mean difference between actual patient body mass and the mass of the phantom chosen to represent the patient, was 17%.

Forty five percent of all patients received more than one scan over the time period of the study; 50% of all patients aged 15 years old and younger received more than one scan, and 25% of patients aged 16 and 17 received more than one scan.

One patient who underwent 10 examinations during the study period received a cumulative eye lens absorbed dose of 81.9 mGy, and the five organs with the highest total dose were the heart, urinary bladder, thymus, liver and brain.

## Discussion

An important first step to managing patient dose in PET-CT is finding suitable methods to quantify dose from both the CT and the PET portions of the examination. Methods which incorporate examination-specific and patient-adjusted parameters require considerable effort to collect and appropriately analyze data but provide results that more accurately represent the individual patient and irradiation conditions than generalized methods. A more accurate result is important for patients who are likely to receive multiple scans over the course of their disease management. As a retrospective investigation, this study entailed extraction of data from electronic records but a future evaluation could reduce time spent locating data in records by manually prospectively recording data such as injected activity, patient data and CT technique at the time of the examination. Our reported results represent pediatric patients in our institution and should be compared to other patient populations carefully. While the dosimetry tools employed in this study utilize phantoms of both genders, the reported results are gender-averaged. It should be noted that because we defined pediatric as less than 18 years old, only patients who were less than 18 years old at the time of exam were included in this study. The limited number of patients aged less than 1 year old in this study does not provide definitive findings for patients in this age group. The 429 examinations for which dosimetry was performed represent 133 unique patients, indicating that patients often underwent multiple scans. About half of the patients in this study had more than one PET-CT scan and 7% had 5 or more scans, supporting the importance of ongoing monitoring of individual radiation dose. One notable patient had 10 scans during the study period and received a cumulative eye lens absorbed dose of 81.9 mGy. While CT doses below 2 mSv are achievable for PET-CT, the average CT dose of 6.4 mSv for the patients in our study reflects the objective of pediatric PET-CT exams at our institution to provide localization information along with attenuation correction from the x-rays.

OLINDA 2.0 represents many improvements over the previous version, which serve to increase the accuracy of individual patient dosimetry. The software employs the latest phantoms of both genders, which are neither voxelized nor stylized, but are anatomically realistic and can easily be modified. Dose coefficients based on older stylized computational phantoms have been found to be different from those based on newer hybrid phantoms, especially for smaller body sizes. As shown in Table 8, dose coefficients provided by OLINDA 2.0 are lower than those provided by ICRP 128. The exceptions are the heart, stomach, esophagus, and thymus for which OLINDA 2.0 estimated a higher absorbed dose per unit injected activity than ICRP 128. Dose coefficients for urinary bladder, kidneys, heart, red bone marrow and lungs were estimated by OLINDA 2.0 to be lower than ICRP 128. Relative differences between ICRP 128 coefficients and those reported in our study are consistent with those demonstrated by Khamwan et al., in which lower lung and urinary bladder dose coefficients were attributed to improved approximation of adjacent organ boundaries as modeled by newer phantoms, compared to older stylized phantoms [32]. As a result of the organ dose differences between the two methods, the ED coefficients also differ, with those estimated by OLINDA 2.0 being approximately 34% less than those provided by ICRP 128. In accordance with ICRP 103 methodology, effective doses are calculated in the softwares by averaging gender-specific dose. Table 7 includes adult organ dose and ED coefficients for reference, with differences in the coefficients being consistent with those in pediatric phantoms. OLINDA 2.0 reported dose factors for left colon, right colon and rectum and we report total colon PET dose as the average of the three. The adjustment in OLINDA 2.0 of phantom organ mass made phantoms more representative of individual patient body size than the default phantom, but still not as specific to the patient as would be from segmentation of an actual patient image. Additionally, modification of all organs by the same ratio does not accurately reflect a non-linear change in organ mass with body mass.

**Table 8:** Comparison of Organ Dose Coefficients, OLINDA v2.0 and ICRP 128

	1Y		5Y		10Y		15Y		A	
	OLINDA	ICRP	OLINDA	ICRP	OLINDA	ICRP	OLINDA	ICRP	OLINDA	ICRP
Adrenals	0.067	0.071	0.038	0.039	0.024	0.024	0.016	0.016	0.014	0.012
Brain	0.058	0.063	0.044	0.046	0.040	0.041	0.038	0.039	0.038	0.038
Colon	0.067	0.070	0.039	0.039	0.025	0.025	0.016	0.016	0.014	0.013
Esophagus	0.077	0.066	0.042	0.035	0.026	0.022	0.017	0.015	0.014	0.012
Gallbladder	0.061	0.070	0.035	0.037	0.024	0.024	0.016	0.016	0.014	0.013
Small intestine	0.066	0.073	0.037	0.040	0.023	0.025	0.015	0.016	0.012	0.012
Stomach	0.062	0.067	0.035	0.025	0.022	0.022	0.015	0.014	0.013	0.011
Heart	0.295	0.380	0.224	0.210	0.165	0.130	0.089	0.087	0.040	0.067
Kidneys	0.060	0.078	0.034	0.045	0.021	0.029	0.014	0.021	0.012	0.017
Liver	0.102	0.120	0.061	0.063	0.042	0.042	0.029	0.028	0.024	0.021
Lungs	0.111	0.120	0.059	0.062	0.037	0.041	0.024	0.029	0.020	0.020
Pancreas	0.069	0.076	0.041	0.040	0.025	0.026	0.017	0.016	0.014	0.013
Red bone marrow	0.044	0.059	0.024	0.032	0.016	0.021	0.012	0.014	0.011	0.011
Ovaries	0.078	0.076	0.043	0.043	0.025	0.027	0.078	0.018	0.016	0.014
Spleen	0.059	0.066	0.033	0.035	0.020	0.021	0.013	0.014	0.011	0.011
Testes	0.061	0.066	0.034	0.037	0.023	0.024	0.014	0.014	0.013	0.011
Thymus	0.069	0.066	0.039	0.035	0.025	0.022	0.017	0.015	0.014	0.012
Thyroid	0.061	0.065	0.034	0.034	0.021	0.021	0.013	0.013	0.010	0.010
Urinary bladder	0.448	0.470	0.243	0.340	0.157	0.250	0.105	0.160	0.093	0.130
Uterus	0.097	0.090	0.058	0.054	0.040	0.036	0.025	0.022	0.021	0.018
ED	0.073	0.095	0.041	0.056	0.027	0.037	0.018	0.024	0.016	0.019

1Y = 1-year-old phantom. OLINDA 2.0 gender average

5Y = 5-year-old phantom. OLINDA 2.0 gender average

10Y = 10-year-old phantom. OLINDA 2.0 gender average

15Y = 15-year-old phantom. OLINDA 2.0 gender average

A = Adult phantom. OLINDA 2.0 gender average

VirtualDose CT software also utilizes the current generation of computational phantoms while offering the ability to incorporate exam-specific parameters. Compared to doses estimated using MIRD-style phantoms, the doses estimated by VirtualDose CT can be higher or lower depending on the location of the organ, but more accurately represent the patient, so are understood to be more accurate [33]. The improved approximation of human anatomy of phantoms in both VirtualDose and OLINDA 2.0 also means the organs represented are not exactly consistent across all ages, so doses from different age phantoms must be aggregated with care. For example, breast dose is only reported for 15-year-old and adult female phantoms, and not reported for 1-year, 5-year, 10-year phantoms of either gender. While VirtualDose reports eye lens dose and OLINDA 2.0 does not, eye lens dose results are included in this study for reference. Due to the accumulation of FDG in the brain, some dose to the eye lens is expected from PET.

Because phantom selection was based on a comparison of phantom mass with patient mass, some pediatric patients were best modeled by phantoms, which did not necessarily correspond to patient age in both PET and CT dosimetry software. For example, several patients were best approximated by adult phantoms. While PET organ dose could be more accurately represented by modification of phantom organ mass by the ratio of phantom mass to patient mass in PET software, it should be noted that CT organ mass was fixed to the chosen phantom. Although all of our pediatric PET-CT exams are conducted without tube current modulation, (TCM) the influence of this technique on patient dose should be considered where it might be implemented, such as a PET-CT examination that includes a diagnostic-quality CT. Failing to account for TCM can result in an over- or under-estimation of dose depending on the body region imaged. When the tube current is modulated, an organ dose estimation method based on a single CT dose metric such as dose length product (DLP) does not accurately represent patient dose, indicating the need for comprehensive dose estimation using appropriate methodology. Anatomy selection and accurate representation of patient size and composition are important considerations for pediatric CT patients, because organ dose changes are relatively greater in smaller patients depending on anatomy selection. A recent study demonstrated organ dose change resulting from inclusion or exclusion of an organ in scan range is more drastic in small patients [34]. In light of the wide range of considerations for accurate dosimetry, including patient size, age and imaging technique, a variety of dosimetry methodologies including those examined in the current study are beneficial to have on hand.

## Conclusions

Radiopharmaceutical and x-ray internal radiation dose adjusted to individual pediatric patients can be estimated with available methods, which utilize appropriate anatomically-realistic models with patient-adjusted inputs. The ability to routinely evaluate dose representative of individual patients is especially important for radiosensitive populations such as children and radiosensitive organs subject to deterministic effects such as the lens of the eye. Dose estimates, whether organ or effective dose, are central to understanding how radiation dose relates to patient detriment and are important groundwork for a rigorous benefit analysis applicable to any medical imaging modality. Organ doses estimated using methodology employing anatomically realistic phantoms can be considerably different from those organ doses based on older generalized phantoms, but are understood to be more accurate because of the anatomical realism. Along with long-term monitoring of disease management outcomes, routine evaluation of individual patient dose is a key component in improving the understanding of the relationship between radiation exposure and biological effect. Whether for justification of examinations, long term tracking of patient doses or optimization of protocols, dose estimates are achievable, which are expediently formulated using appropriate methodology that closely represents the patient. As truly patient-specific dosimetry is becoming more and more achievable, patient-adjusted methods such as those in the current study facilitate a meaningful understanding of patient radiation dose by accounting for dosimetry factors representative of the patient and exposure scenario.

## Data Availability

The datasets used and/or analysed during the current study are available from the corresponding author on reasonable request.

## Abbreviations

18F-FDG-2: 18-Fluoro-2-deoxy-D-glucose
AAPM: American Association of Physicists in Medicine
ACR: American College of Radiology
BSA: Body Surface Area; CT-Computed Tomography
CTAC: CT Attenuation Correction
CTDIvol: Volumetric Computed Tomography Dose Index
DLP: Dose Length Product
EANM: European Association of Nuclear Medicine
ED: Effective Dose
ICRP: International Council on Radiation Protection
MIRD: Medical Internal Radiation Dose Committee
PET: Positron Emission Tomography
TCM: Tube Current Modulation;
